# Emotional Experiences of Obese Women with Adequate Gestational Weight Variation: A Qualitative Study

**DOI:** 10.1371/journal.pone.0141879

**Published:** 2015-11-03

**Authors:** Débora Bicudo Faria-Schützer, Fernanda Garanhani de Castro Surita, Vera Lucia Pereira Alves, Carla Maria Vieira, Egberto Ribeiro Turato

**Affiliations:** 1 Department of Gynecology and Obstetrics, School of Medical Sciences, State University of Campinas, Campinas, São Paulo, Brazil; 2 Clinical Qualitative Research Laboratory, School of Medical Sciences, State University of Campinas, Campinas, São Paulo, Brazil; 3 Department of Medical Psychology and Psychiatry, School of Medical Science, State University of Campinas, Campinas, São Paulo, Brazil; 4 Department of Nutrition, Methodist University of Piracicaba, Piracicaba, São Paulo, Brazil; University of Helsinki, FINLAND

## Abstract

**Background:**

As a result of the growth of the obese population, the number of obese women of fertile age has increased in the last few years. Obesity in pregnancy is related to greater levels of anxiety, depression and physical harm. However, pregnancy is an opportune moment for the intervention of health care professionals to address obesity. The objective of this study was to describe how obese pregnant women emotionally experience success in adequate weight control.

**Methods and Findings:**

Using a qualitative design that seeks to understand content in the field of health, the sample of subjects was deliberated, with thirteen obese pregnant women selected to participate in an individual interview. Data was analysed by inductive content analysis and includes complete transcription of the interviews, re-readings using suspended attention, categorization in discussion topics and the qualitative and inductive analysis of the content. The analysis revealed four categories, three of which show the trajectory of body care that obese women experience during pregnancy: *1) The obese pregnant woman starts to think about her body;2) The challenge of the diet for the obese pregnant woman; 3) The relation of the obese pregnant woman with the team of antenatal professionals*. The fourth category reveals the origin of the motivation for the change: 4) *The potentializing factors for change*: *the motivation of the obese woman while pregnant*.

**Conclusions:**

During pregnancy, obese women are more in touch with themselves and with their emotional conflicts. Through the transformations of their bodies, women can start a more refined self-care process and experience of the body-mind unit. The fear for their own and their baby's life, due to the risks posed by obesity, appears to be a great potentializing factor for change. The relationship with the professionals of the health care team plays an important role in the motivational support of the obese pregnant woman.

## Introduction

The obese population has increased systematically worldwide in the past few decades [1]. Obesity is considered a multifactorial and complex health problem [2], [3]. From a health care professional standpoint, despite a few disagreements, obesity is predominantly characterized as an illness [1].

As a part of the obese population, the number of overweight/obese women of a fertile age has also increased [4]. This phenomenon is a current challenge for health services since pre-pregnancy obesity and/or excessive gestational weight gain may harm both mother and baby [5], [6], [7]. On the other hand, a pregnancy is a moment of huge physical, psychological and social changes in the life of a woman. A woman’s physical and emotional health are inseparable aspects at this particular time [8]. Pregnancy is also a mark of emotional development in a woman [9] and is frequently accompanied by behavioral changes and positive attitudes towards weight and nutrition [10].

The obstetric outcomes may be unfavourable when related to obesity in pregnancy. The following have been cited: gestational diabetes, hypertensive syndromes of pregnancy, prolonged labor, surgical delivery, macrosomia, fetal distress, cephalopelvic disproportion, trauma, asphyxia, neonatal death and prematurity, oversized fetus for gestational age[11,12]. Compared with women of normal weight, obese women run a greater risk of death, not just in adulthood but also in the gravidic-puerperal cycle. According to recent data from the UK, 35% of all cases of maternal death were of obese women compared to 23% of the general maternal population—a dramatic increase when compared to the 16% mortality rate in 1993 [13].

To ensure the health of mother and baby, since 2009 the current guidelines of the Institute of Medicine [14] include a specific and relatively narrow margin of gestational weight gain for obese women in the pre-gestational period. The characteristics of obesity require comprehensive studies that go beyond the biological aspects, principally because studies on obesity, particularly during pregnancy, have been mainly focused on the diet and physical activity binomial [1], [3]. Even though psychosocial factors such as depression, anxiety, stress and low self-esteem are associated with obesity during pregnancy [4], [15], [16], [17], [18], the number of researches that focus on the psychological aspects of an obese woman’s pregnancy are scarce [15], [16], [18]. Molyneaux et al [18] affirm that the impact of obesity on physical health during pregnancy has been studied extensively, while the relationship between obesity and maternal mental health has been largely neglected. Based on their review, women who are obese when they become pregnant are more likely to experience elevated antenatal and postpartum depression symptoms than normal-weight women, with intermediate risks for overweight women [18].

According to Furness [19] in a study of maternal obesity support service, psychological factors were: Self-talk: they would use their pregnancy as an excuse for overeating and covering up previous eating disorders and were able to realise that this internal dialogue was not realistic. This suggests that some input from health professionals responsible for prenatal care may help obese women cope with these internal messages and support them so that they can make the necessary changes at an early stage. Motivation is the second point: the obesity women have to wage an enormous struggle to find motivation to exercise, change eating habits, and maintain a positive attitude for a long period of time. Finally, he points to Social support as another main element, as women with obesity reported feeling lonely and isolated at times. The support of others motivated them to eat well and become more physically active.

The feelings and experiences of obese pregnant women were also studied by Furber [20] and Nyman [21]. They emphasize the importance of consistency and continuity in the support given to the obese woman during pregnancy by the health team. They affirm that feelings of humiliation and judgment/scrutiny are experienced during their pregnancy, even when doing physical exercise.

In this sense, pregnancy presents itself as an opportune moment for the intervention of the health care team with regards obesity. Since during this phase of their life, women can accept their weight and corpulence better, are closer to the health care team and have more opportunities to acknowledge the benefits of a healthier life style for themselves and their future baby [22]. Nyman [21] states that the obese pregnant women perceived pregnancy as the only moment in which admitting and revealing their excessive weight was acceptable.

Therefore, considering that pregnancy is a privileged moment in the life of a woman and mainly of an obese woman vis-à-vis the greater attention they can give themselves, the general objective of this study is to describe the connection established by the interviewees, between the emotional experiences of pregnant women with pre-gestational obesity and the motivation in keeping an adequate variation of weight during while receiving prenatal care. Based on the analysis of the content conveyed by the interviewees, it is hoped that the results of this work serve to improve the understanding of affective and psychological aspects of obese pregnant women, based on the premise that by providing health care teams involved in ante-natal care with appropriate tools, they in turn will be able to deal more competently with the obese pregnant woman, offering conditions that favor their adherence to health service advice and thus also contribute to postnatal maternal health.

## Subjects and Methods

### Study design

The intention was to describe the experiences of women who managed to maintain an adequate weight gain in the belief that the understanding of this process could be a way of empowering the health care team in their care for all those patients with the same condition, thus expanding the care given to this specific group.

The understanding of the experiences and consequently their description is based on the precept that it could be achieved only through disclosing the meanings attributed by the individual herself. Therefore, to attend the researcher's interest in the meaning qualitative research is usually developed because it facilitates the grasping of the meanings attributed by those who go through a given experience. In this method, the individual interviews are considered a research tool that strives to study the descriptions of the lived experience. [23].

Therefore, centered on the interviewee’s discourse, the scientific investigation seeks to capture the meaning the subjects attribute to their lived experiences, based on the conjecture that this is an efficient way to learn and to infer results that reveal coherence in meaning [24], [25], [26].

Thus, in order to grasp meanings attributed to experiences within the health/illness context, generally speaking this methodological philosophy is transposed by means of a posture that is analogous to clinical practice. In Brazil, some researchers consider that the qualitative design of research acquires a clinical-qualitative perspective when applied to the health area and thus denominate their studies [27], [28], [29]. The clinical-qualitative perspective proposes the investigation of meanings that individuals give to their life experiences while receiving health care. With a view to this, the interviews are usually individual because they had been devised to capture the lived experience in depth.

In the context of health, the data collection would take place in the natural setting of the interviewees, that is, in the place where the patients are involved in their clinical processes. Thus, the researcher needs to become familiar with its functioning, and to do so, he takes notes of conversations with the professionals and of the expectations of the patients with this clinical condition. These notes could be useful to clarify and to better understand the descriptions of experiences related by the interviewees. Thus, the researcher's perceptions and accounts of the conversations with the professionals and pregnant women are registered. This kind of notes and our first knowledge of the context to be researched begins to show us to what to pay more attention to during the interviews. The construction of the sample in qualitative studies was deliberate, with people who were carefully chosen according to the theme of the research and who were considered as social representatives of the situation being studied [28], [29], [30], [31], [32].

### Research environment

This study was conducted in a public hospital in the interior of Brazil. The hospital is a reference in women’s and neonatal healthcare and has a multi-professional and interdisciplinary team as well as fomenting teaching, research and further education. It covers a region of 100 municipalities, carrying out 82.000 outpatient consultations per year. The hospital has two prenatal out-patient clinics: the high risk antenatal clinic (PNAR), which cares for pregnant women referred due to their clinical condition or obstetric risk; and the specialized antenatal (PNE) which cares for high-risk pregnancies and vulnerability, after an initial triage in the PNAR. The present study, considered to be extremely important by the health team care from this service, was carried out in one of the outpatients’ clinics where pregnant women with obesity, hypertension, rheumatic, renal, hematological and oncological diseases are attended. In this setting, all those involved were informed about the presence of the researcher and the subject of the research. The research has conducted throughout 2012 and 2013.

### Recruitment and participants

The criteria for inclusion in the sample of the present study were: pregnant women over 18 years of age; around 34 weeks of gestation or more (in order to guarantee that they were at the end of their pregnancy); a clinical profile of pre-pregnancy obesity (IMC ≥ 30 Kg/m^2^); receiving treatment at the specialized high-risk prenatal care outpatient clinic of the hospital; with a gestational weight variation within the limits recommended by the Institute of Medicine [14] for obese expectant mothers. Pregnant women who lost or maintained their weight during pregnancy were evaluated by the doctors of the clinic and were found to be clinically well due to the absence of maternal-fetal harm related to this issue.

With regard to the closure of the sample, the criterion of theoretical saturation of information was used. The theoretical saturation of information is clearly defined and it represents the number of conducted interviews after which reports become repetitive, bearing in mind each category is being designed in an on-going process: the data is being treated simultaneously in a process developed together with the advisor and validated by peers of the research group. In addition, new interviews would not bring other significant data, given that the categories for discussion had already achieved consistency, thus complying with the initial objectives [33]. The recruitment of participants took place between October 2012 and June 2013.

### Data collection

The interviews took place between January and June 2013.The researcher and the medical team made a prior selection using the patients’ medical files from among those who fulfilled the inclusion criteria. On the day of the patient’s antenatal medical consultation, the researcher approached the women and invited them to take part in the research and to give a recorded interview. All the women invited accepted the invitation, with no refusals, which revealed the emotional availability of the women to address the issue.

Based on the expectation that the interviewees would speak as openly as possible about their experiences in relation to aspects of weight during pregnancy, semi-structured individual interviews with the open question were used to collect the data [26]: *Tell me about how you have been feeling about weight during this pregnancy*?

The interview that began in this way was planned based on a script with questions that were determined by the aim of the study and that provided support for the researcher:

- Have you thought or done anything about your weight during this pregnancy?
- What were your motivations?
- What do you think causes someone to put on weight?
- Tell me how you felt during the antenatal appointments here in the outpatient clinic?
- What have you thought about the baby that is going to be born?
- What do you feel about the professional team that is looking after you here in the outpatient clinic?

These were more cues than questions that were used in the appropriate moment of the interview, if the interviewees had not broached these topics spontaneously.

Thus, these women were able to speak as openly as possible about their experiences, enabling the subsequent discovery of the meanings they gave to certain existential phenomena connected to the adequate variation of weight while pregnant and obese [34]

The interviews, lasting an average of 45 minutes, were carried out by the first author of the article (DBFS) in a suitable office in the outpatients’ clinic. The participants spontaneously agreed to give the interviews. After establishing a *rapport*, the theme and objectives of the research were explained, the Informed Consent was signed and the identifying information of the interviewees was collected. An authorization to use a recording device was requested. All the interviews were taped.

Notes were also taken, unsystematically, about the non-verbal expressions of those interviewed. These had the aim of clarifying or confirming the analysis of the verbal expressions, such as notes on a tear shed or on the tone of voice when addressing a specific theme.

### Data analysis

**Fig 1:**
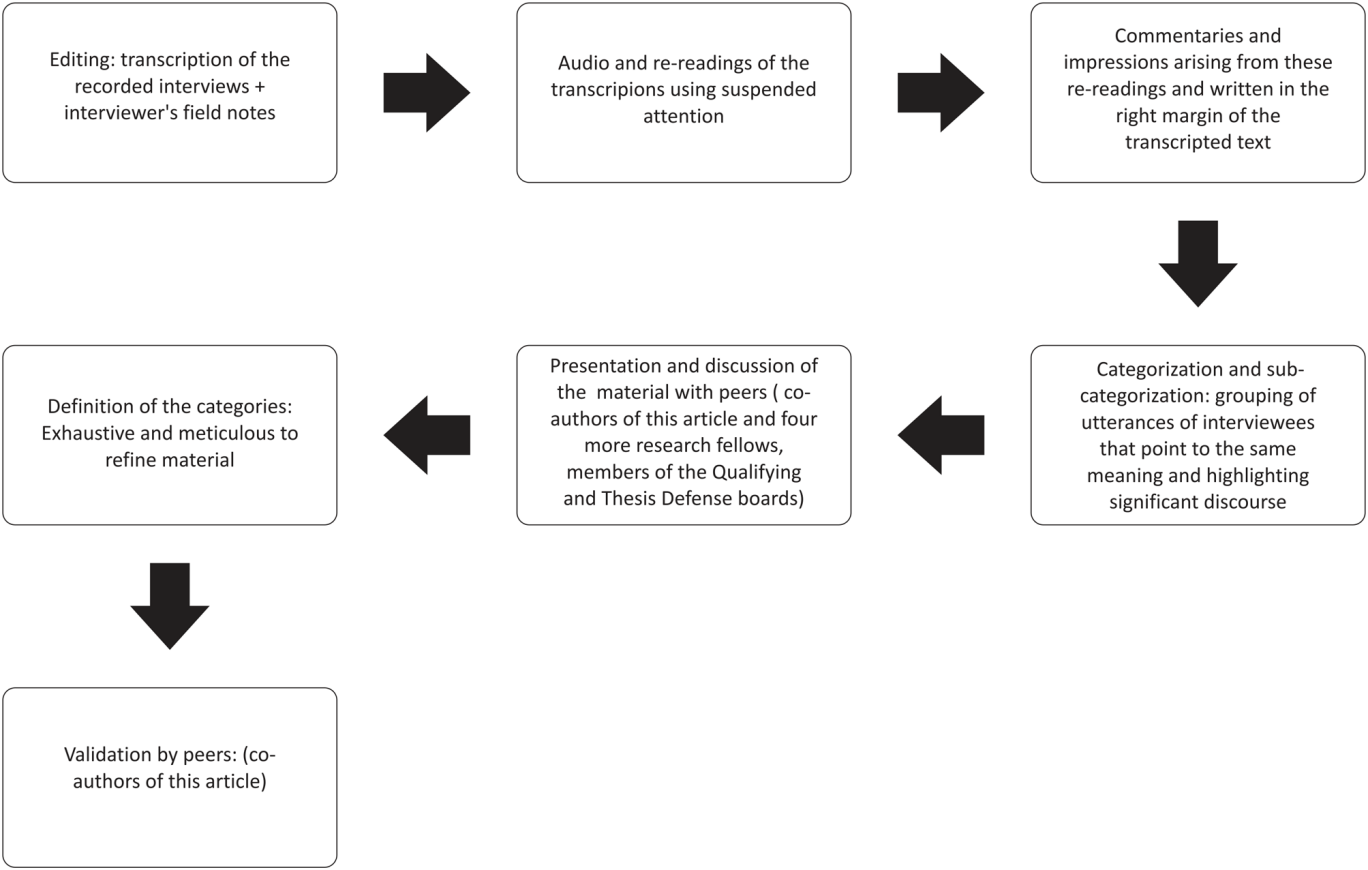
Flow Chart of the Content Analysis.

The complete analysis of the transcripts with the notes written by the researcher comprise the material of the study, which meant that the main researcher had to edit this, as can be seen in the flow chart (Fig 1).

The set of the transcribed text of each recorded interview contained 97 pages that comprised the material for study of this research, analyzed in accordance with the Content Analysis developed by Bardin [35]. To comply with Brazilian Law, this information is sealed.

**Fig 2:**
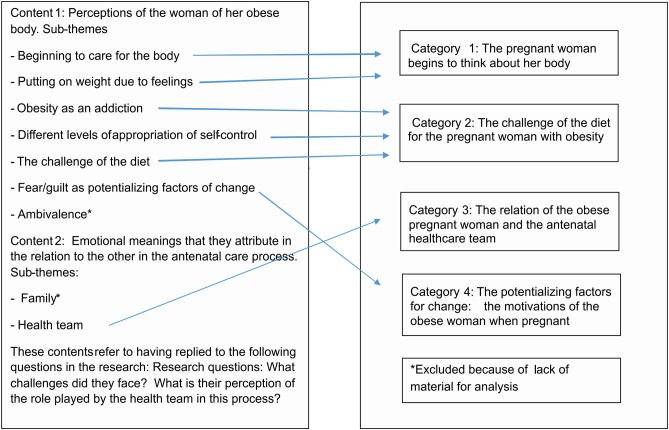
Process of constructing categories.

The technique of Content Analysis was used to handle the data, consisting of a set of analysis techniques that allows the replication and validation of inferences about research data within a scientific context [34], [35], [36], [37], [38], [39], [40], [41], [42]. An inductive process was adopted in which the categories and the explanatory hypotheses were formed, based on the data collected relating to the aims of the research. Firstly, the interviews and the researcher's observations were fully transcribed. Then, a reading of the material using suspended attention was performed, allowing the invasion of impressions and activities so as to suspend directed attention to the maximum [36], [43], [44], [45]. The next step was the selection of the units of meanings guided by the questions that the research would answer. The following phase corresponded to the categorization work [27], [43], [44], [45], in which the issues were pointed out according to their level of intimacy or proximity, organizing the themes that expressed important meanings and elaborations and that met the objectives of the study to produce new knowledge [37], [43], [44], [45]. The validation of the findings of the material was given by peers from the Clinical Qualitative Research Laboratory of the Medical Sciences Department of the State University of Campinas and through national and international scientific events. The process of data analysis and its relation with the questions of this research are explained in Fig 2.

### Ethical approval

This research was approved by the Ethics Committee of the School of Medical Sciences of the State University of Campinas and took into account the requirements laid down by the Brazilian National Board of Health. All participants signed the Informed Consent before the interviews.

## Results

Thirteen interviews were conducted for this study. The average age of the interviewees was 32 years; eleven of them were in a partnership and two others had no partner. Parity varied among the pregnant women. Three were primigravida, two in their second pregnancy and five were in their third or more pregnancy. Other bio-demographic characteristics of the interviewees are recorded in Table 1.

**Table 1:** Biodemographic characteristics of the subjects, 2013.

Participants*	Pre-pregnancy IMC**	Gestational weight variation (Kg)
1	31	+6
2	40	+9
3	35	+5
4	48	+7
5	52	+4
6	53	0
7	39	-2.3
8	65	-1.75
9	31	+6.5
10	33	+6.3
11	33	-4.2
12	42	+0.9
13	52	+4

*The order of the table refers to the chronological order of the interviews.

**The IMCs were calculated on the pre-pregnancy weight declared by the interviewee and considered from the analysis of the initial weight at the beginning of the monitoring of the service.

When these 13 women were invited to talk about their experience of successful weight control, they showed interest and availability. During the interviews, they spoke as freely as possible about their experiences and feelings regarding their obesity and the pregnancy. Thus, the results reported here emerge from content analysis developed from the text of the interviews.

Analyzing the motivations and their emotional significance in bringing about changes in health habits and self-care is complex and far-reaching. The results of this research show that a challenge was posed to these women, one that goes beyond changes in behaviour. It is an existential challenge of finding the motivation to generate changes in themselves. The text analysis of the interviews shows us various aspects of great importance for understanding the experience lived by these women. They are aspects separated into four thematic categories. Three of these show the trajectory of body care that obese women experience during pregnancy: 1) The pregnant woman begins to think about her body; 2) The challenge of the diet for the pregnant woman with obesity; and 3) The relation of the obese pregnant woman and the antenatal health care team. The fourth category reveals the origin of the motivation for the process of caring for themselves: 4) The potentializing factors for change: the motivations of the obese woman when pregnant

S1 Fig shows the categories found, based on the interviewees’ discourse as well as a synthesis of the results, highlighting the main findings.

### 1) The pregnant woman begins to think about her body

The interviewees, when reflecting about adequate weight variation, associate the act of eating with their emotions:

*I think it is my anxiety [that makes me put on weight]*. *I am the most anxious among the children*, *the most concerned*, *electric*… *I think it really is anxiety (Interviewee 2)*.

*I think anxiety is what makes people gain weight*. *If you are anxious about something*, *or worried*, *you eat*, *sometimes you aren't even hungry*, *but you eat and it seems that chewing calms you down*. *That's what it's like for me*: *I need to eat to calm down*. *(Interviewee 3)*

This account shows how the interviewees associate obesity with emotional conditions, such as: anxiety, preoccupation, nervousness, and stress. When saying that the act of chewing calms her down, interviewee 3 reveals a difficulty in exerting self-control that is concomitant with the efforts she makes in the attempt to contemplate her body. The women interviewed in this study showed that when they managed to look at their obese bodies during pregnancy, they were also able to start thinking of their manner of being and feeling.

… *you have to convince yourself that you have to do this*, *because those who like to eat*, *like to eat*… *you have to first get it into your head and then into your body (Interviewee 6)*

The relationship between mind and body, as shown by interviewees is an aspect to be taken into consideration in dealing with these patients. They simultaneously show that these aspects tenuously begin to be experienced as bearing a relationship with each other “first the mind and then the body” and point to a possible integration. This was also exemplified by interviewee 6 when she verbalizes the relationship between her feelings and her desire to eat.

They therefore explained that weight control and the adherence to the diet are closely connected to psychological components. This integrating experience allowed them to realize that making new choices is possible, with the support, attention and reception of the team in order to adhere to new dietary behaviour.

*Now*, *when I am about to eat I remember the doctor*. *If I eat she will scold me*, *so I try to eat less*. *Before*, *I didn't have such control*, *I was so anxious*, *that I would eat anything in front of me*. *(Interviewee 4)*

### 2) The challenge of the diet for the pregnant woman with obesity

The adoption of new dietary behaviour and the fact that they see themselves as responsible for their own care enabled them to feel victorious and transformed. At the same time, a history of failures, certain unfavourable mental states and the desire to eat are daily challenges for these women.

… *the diet is a challenge for people who have been in that momentum of eating*, *anxiety and then suddenly have to close their mouths*. *It's hard for those with obesity*. *Saying that dieting is easy*, *it's not*, *you have to go hungry a lot*, *even with a balanced diet*, *I have cravings (Interviewee 12)*

The interviewees of this study reported feelings of sadness, anger, fear and a struggle against the “addiction to food”.

… *I have no control*. *If I eat something sweet I want something salty*, *if I eat something salty I want something sweet*. *If I drink water*, *then I want a soda (Interviewee 8)*

The interviewees were able to report their hesitations and the challenges they face with respect to the diet and the addiction as well as their responsibility and control over their life style. Encouraged by the challenge of the diet and the awareness of their obese body and the consequences for their and their baby's health, the expectant mother is faced with a dilemma: adhere to the diet and habits that will bring a healthier life style or continue to be passive when faced with her feelings and the control of her own life? The results of this research show that the women who were interviewed were able to establish dietary practices that promote self-care, thus confronting their hesitations and habits. As shown by the fragment from interview 8, during the interviews one perceives the daily challenge and struggle to take control over their desires

The discourse of interviewee 4 shows that the relationship with food can be similar to an addiction to drugs. This shows the peculiarities in these women’s behaviour in regard to the challenge of adhering to the diet in an attempt to release themselves from a subjective condition of slavery to food and when faced with the desire of finding alternatives to cope with their inner discomfort.

*I started working and my self-esteem improved and instead of making me eat less*, *I ate more*. *Instead of thinking that I wouldn't eat in order to lose weight*, *to be healthier and better*, *that's when I ate*. *It is something stronger*, *compulsive*… *it is hard to lose weight*, *control that anxiety*, *that desire to eat when you can't eat*. *I think that it's like a drug addiction*, *it is a food addiction (Interviewee 4)*

It is important to point out that the results of this study indicate that despite having a higher level of responsibility for their actions, the interviewees somehow attributed this change more to the fear of something happening to someone else (an external agent) and not to themselves

*“it's not just us*, *there is the baby too*…*but because I was pregnant” (Interviewee 12)*.

Findings in this study that indicate the great challenge experienced by the pregnant women interviewed when looking at themselves, acknowledging their own body and daily behaviour, recognizing their conquests and difficulties as well as changing the manner they relate not only to food but to their life:

*“Nowadays I find it easier to deal with nutrition*. *Eating a salad*, *a stew is much healthier than eating junk food*, *you see*. *I know that that is good for me and for the baby*. *That is how it is today*, *I deal better with this*…*but in the beginning it was hard*…*It was a change in mind and body*. *Really*, *an overall change*. *Doing it all was worthwhile*, *because up to that moment*, *I didn't know how to deal with it*.*” (Interviewee 11)*

The adoption of new manners of eating, supported by dietary instructions, brought significant consequences to these women's lives. One result that this study outlines is that some of the interviewees lost or maintained their weight during pregnancy without harming their health or the baby's health, which may be connected to the change in eating habits. These results show how much they were motivated to do this.

### 3) The relation of the obese pregnant woman and the antenatal health care team

The topic of the relationship between the interviewed expectant mothers and the antenatal health care team permeated all the accounts. The analysis of the accounts revealed that the monitoring and prenatal care through medical and nutritional consultations, routine exams and nursing care were opportune occasions to encourage these changes.

Positive references about the relationship with the service and the prenatal care professionals were seen in the interviews. They had an important role in the sense of helping them look at themselves and face their anxieties, giving them support in their hesitations and difficulties.

*Here I improved*, *because here I can talk*. *Every appointment you go in*, *you talk to them*. *I have a good weight but they will scold me because I gained 1 Kg in 14 days*. *I think if there is someone there talking*, *talking*, *explaining*, *showing*, *it helps a lot (Interviewee 4)*

The health care team is once again perceived as an agent that is important for the support of the psychological strengthening of these women when facing their troubles and in these experiences in question. Support from other people motivates them to eat in a healthier manner and become more active, which contributes to their more complete adherence to the new attention to their nutrition, among other therapeutic and preventive health measures. The interviewees showed that the manner in which the professionals approach the issue of obesity is very meaningful to them.

The routine of consultations, exams, admittance to hospital and concerns about weight gain were perceived attentively and with extreme care by the interviewees. The attention and support that the healthcare team can provide is an important tool in dealing with anxiety, guilt, feelings of despair and the inability to control their weight. It can be seen that the attention and care of the professionals can favour the development of attention and self-care in the obese pregnant woman. The continuity of this care by the same professionals was another aspect valued by them, as well as clear warnings about pregnancy and weight, which helped these women to alter their behavior, mainly in relation to eating habits.

*…I was seen about six times by the same doctor*, *she’s so nice*, *she cleared up all my doubts (Interviewee 8)*

*They transmit a great deal of assurance*, *the nurses are wonderful*, *the first time I came they explained about snacks*…*they tell us to rest*, *it’s really good (Interviewee 9)*

*…the doctor who I have seen from the beginning until now is marvelous*, *the nutritionist’s team is also good and the nurses*… *here you do all the exams*, *you do everything that has to be done*, *you are admitted to hospital*,…*they really go deep into it*,…*it’s a team that works to help you during pregnancy (Interviewee 11)*

The interviewees refer positively to the care offered, relating experiences that favored dialogue and an atmosphere of full care by the multidisciplinary antenatal team. However, some of those interviewed complained about the turnover of doctors in this service and spoke about the importance of being cared for by the same doctor so as to develop trust and closeness.

The analysis of the results showed that caring fully and continuously for these women allows more genuine dialogues and important insights for the pregnant woman, besides guiding the team’s follow-up conduct for that particular woman.

…*They go deep*, *you see*? *They want to know everything*, *even what I ate in the morning*! *Their care was really good*, *I think they stopped my blood pressure from rising*, *they are really caring*, *I’m in good hands (Interviewee 10)*.

### 4) The potentializing factors for change: the motivations of the obese woman when pregnant

The interviews also gave us the possibility of discovering and describing an additional element that could be the central element of this lived experience of good weight gain; the potentializing force in the process of managing the gestational weight variation.

When approached as to the motivation for the adherence to dietary recommendations and to a self-care process during the pregnancy, the interviewees reported feelings of connection to their own lives and their baby's. They pointed out that the attention to the pregnancy and the baby was motivated by the fear that the risk caused by obesity could harm their future child or even lead to the mother's death.

*I’m thinking a little more in this pregnancy because of the delivery*, *fear*, *because if I put on a lot of weight*, *my blood pressure rises; I’m managing to control this more out of fear (Interviewee 5)*

*I have to be conscious that it’s not just me*, *right*? *There is another life*, *too*. *You are creating another life*…*so*, *you have to be careful because it can cause various problems*… *(Interviewee 7)*

…*I am managing*…*it's too much risk*, *I try to avoid it*. *And it's not just us*, *there is the baby*…*but it's because I am pregnant*…*what if I gain weight and it gets worse*. *My oldest son loses his little sister and me*, *right*? *So*, *I prefer to avoid it (Interviewee 13)*

The interviewees’ fear and guilt generates the possibility to repair possible damage caused by the obesity through the adoption of new health habits and care. The findings of this research show how important it is for the health care team to identify these feelings in order to help these women to experience a guilt that generates positive changes and does not reinforce stigmas connected to obesity and a negative feeling of self-blame.

The account of interviewee 11 supports the issue of fear of death and its acknowledgment:

*The Doctor was very clear; if you don't take care of yourself the baby can die in your belly and you won't even notice*. *It was a shock and that is when I started doing everything right (Interviewee 11)*.

And interviewee 1 expresses the experience of guilt:

*Imagine being guilty about some future problem your child may have that you could have avoided*?! *(Interviewee 1)*

Even if the issue of death is not pointed out by the health care team, one notices that it is a very significant aspect to bring about change. It is a recurring theme in the accounts of the interviewees and the fear of death mobilizes change.

The fragment from interviewee 12 shows an attempt of the interviewees to avoid a bigger torment, something irreparable during the pregnancy. They report that they felt moved by the information about the risks of a pregnancy while obese, which generated the fear of death: theirs and the baby's. For these women the recognition of a pregnancy with a potential for death or irreparable damage to their own health or the baby's served as a trigger for changes in their dietary behaviour and in the care they take with their health in general.

The possibility of getting in touch with the obese body during pregnancy, together with the capacity to tolerate the distress connected to the fear and guilt of causing the death/loss of themselves or the baby, enabled these women to restrain their desire to eat certain foods or quantities and to adopt a new life style during the pregnancy.

## Discussion

There are countless external and internal factors that can influence changes in the habits and self-care obese women have *vis-à-vis* the adequate control of weight during pregnancy. This study shows that pregnancy is a moment in which a woman experiences a transformation in her body that allows her to rethink the links between their mind and body. For an obese woman, this period constitutes a great challenge to go on a diet. However, these interviews showed them to be motivated to overcome this challenge. They were revealed to be motivated by the fear of causing irreparable harm to themselves and to their baby They were also seen to have been influenced by the reception they received from the healthcare team.

Pregnancy is reported as an opportune moment for these women to get more in touch with themselves and their bodies and therefore notice their emotional conflicts more accurately, which might have gone unnoticed if not for this condition. Through the transformations of their body, caused by the physiological changes of this phase, the researched women indicated that they could start a more refined self-care process and experience of the body-mind unit.

The feeling of fear for their own and their baby's life, caused by the risks posed by obesity, is referred to as a great potentializing force for change, enabling these women to choose to change their life habits during the pregnancy. At the same time, the relationship with the health care team plays an important role in the motivational support of the obese expectant mothers and the emotional issues they have that are connected to obesity.

Adequate gestational weight gain is a challenge for any woman and it is extremely necessary for the satisfactory development of the pregnancy [14], while concomitantly pregnancy is a moment of both physical and behavioral transformation in a woman [10]. Studying the connection between these two life and health conditions in a woman became fundamentally important to understand this specific population as well as to empower the healthcare team. The specificity of the clinical condition of obesity when experienced together with the psychosocial factors present in pregnant women, as shown in this study cannot do without a dialogue with the rich scientific literature on two topics. Psychodynamic theories and current scientific literature offer many concepts to underpin the results achieved.

The interviewees in this study make it clear that they are aware that something in their emotions, sensations and anxieties was connected to the desire to eat and that this desire was not necessarily organic in nature as is hunger. When they realized this process and could name it, the women felt challenged to face up to themselves in an attempt to develop greater self-control. In relating the act of eating with their emotions, the interviewees showed that a complex psychic process of psychosomatic integration was taking place. As referred to by Figueiredo [46] *“a mind that*, *so to speak*, *gains the thickness*, *opacity and dynamics of a body and a body that*, *on the other hand*, *is mentalized*.*”*

By associating the act of eating and obesity to a mental aspect, these women brought to light a theme that has been investigated by philosophy, anthropology and psychology over the past centuries: the relationship between mind and body. For Merleau-Ponty the body is exactly what embodies the subject in the world, mediating the relationship of the subject with herself [47], [48], [49]. As shown in the results of this research, the physical and biological changes arising from the condition of pregnancy and the relationship with the antenatal healthcare team presented a propitious moment to obtain new understandings of themselves.

Weight control and keeping to the diet, as shown by the results, constitute a great challenge to the obese pregnant women, involving an internal struggle to find and maintain the motivation both to take care of herself and her baby. These aspects coincide with the qualitative studies of Furness [19], who states that obese expectant mothers report a great struggle to find motivation to exercise, change dietary habits and maintain positive efforts for a long period.

The interviewees of this study reported the same negative emotions, such as: feelings of sadness, anger, fear and a struggle against the “addiction to food” also found in the Nyman study [21]. These aspects bring to light the possibility that the obese body is a manner to express their desires, dissatisfactions and contradictions [50], [51].

The Nyman study [21] shows that obese pregnant women are aware of their obese “pregnant” bodies and reveal a desire to lose weight, while at the same time mentioning hesitation about their capacity to lose weight. The results of this study are similar to those mentioned above inasmuch as the interviewees related their doubts, fears and efforts in relation to the “eating addiction”. This can be understood in accordance with some postulations from McDougall [50] about addiction.

McDougall [50] describes a passive attitude of the addicted subject with regards the object of their compulsion. There is an internal struggle in which the addicted subject is submerged. It is a situation of slavery, not only to the external other, but also especially to the “other” inside themselves. Corroborating the author’s [50] proposition, Goodman [51] proposes a definition of addiction as a process in which a behaviour may function both to produce pleasure as well as provide relief to an internal discomfort.

Another issue raised by the interviewees is their attempt to take responsibility for, and control over, their lives. This is an issue that has been referred to as *Locus of control*: a construct developed to describe the beliefs of individuals about the main underlying causes of the events of their lives. This concept has been progressively broadened, to a point where some authors present it in connection with a cognitive, perceptive and motivational orientation, while others adopt a phenomenological and existential perspective [18], [52], [53].

Literature shows that pregnant women classified as obese have higher *scores* for *locus of external control*, that is, the belief that external factors control their lives; and lower scores for *locus of internal control*, which refers to the belief that they control the events in their lives [4].

Upon reflection on the success in the weight variation, the results of this research are consistent with the literature, indicating that the interviewees assumed a certain responsibility for taking care of their own health and controlling the weight gain. Moreover, the interviewees attributed this change more to the fear of something happening to someone else (an external agent) than to themselves.

McDougall [54] states that the routes for addictions are known to all human beings, in the tendency we have to escape the psychic pain derived from the frustrations of life with replacement paradises. There are those who use objects (beings or things) as the object where everything that seems too difficult to consider as being part of themselves can be unloaded. In this sense, they compulsively search for objects that may serve as depositories for their mental state. With the possibility of perceiving the tendency to blame something else for their own issues, several efforts and struggles are needed to conquer the compulsion to this tendency.

Both the concept of addiction formulated by McDougall [50], as well as the *Locus of external control*, explain the great challenge experienced by the interviewed pregnant women when looking at themselves.

The health care team is once again perceived as an agent that is important for the support of the psychological strengthening of these women when facing their troubles and in these experiences in question. The perceptions of the expectant mother that the health care team may offer attention and support could be an important tool when she is faced with the feelings involved in weight control.

This need for support and attention provided by the team has a role that may be understood in an analogy to the concepts presented by Winnicott. The author shows the concept of holding based on the mother-baby relationship [55], [56]. The theoretical dialogue presented here in connection with this author’s ideas should be understood as expressing the maternal function that the team can develop in caring for the pregnant woman, in the sense of supporting and caring for the mother so that she supports and cares for her baby. Thus, it is proposed that the concept of maternal function should be amplified, transferring it to other relational contexts.

For Winnicott [56], [57], the mother, or whoever exerts a maternal function, is a provider of care for the baby and may offer *“holding*,” a support to the little human being. It is the manner in which the mother treats the baby and worries about the baby, as well as the pleasure she has in taking care of the baby that is shown through the way she holds it, envelops it. *Holding* ensures a continuous presentation of the world to the baby, which is the basis of the feeling of continuity of being, of trust in the world and of personal existence [55], [57]. When comparing maternal care of the baby and that given by the health care service to the obese pregnant woman, it can be said that the team developed this *holding* with these expectant mothers.

In the scope of the emotional meanings of this behavior, Winnicott [55], [56] postulates that the continuity of the being is ensured by the consistency of the maternal care of the baby. It is the consistency of good maternal care that enables the child to experiment “a continuity of being,” that supports their feelings of being real and of always being the same through different experiences. This feeling is the base of *oneself*, it is like the centre of gravity of the being [57]. Thus, in the sphere of the life experiences of pregnancy and obesity, the importance of the prenatal healthcare team’s consistency and continuity for these women can be understood as being similar to the function of the experience of the baby in relation to their mother as described by Winnicott. Throughout the antenatal period, the antenatal healthcare team can offer security and trust for the obese pregnant woman, so that when she perceives the positive feelings of the healthcare team, she can feel better, more confident and secure.

The relation with the professionals during the antenatal appointments has shown itself to be of great importance in keeping up the motivation of these women in relation to weight control. This is corroborated by the literature that says the relationship between obese pregnant women and their health care team may be a facilitator in this process and a guide for these women during the process of taking care of their body [21], [22].

The consistent care, with clear and non-judgemental warnings, was appreciated by our interviewees as was the case in the findings of the qualitative study with pregnant women carried out by Furness [19]. The present study also corroborates other findings of this same author in which he reveals the importance of the consistency and continuity of prenatal care for obese pregnant women. The continuity of care by the same professionals favours the development of a relationship, and clear messages about pregnancy and weight help to modify their habits. In the ambit of the emotional meanings of this behavior, Winnicott [56], [57] postulates that the continuity of being is assured by the constancy of the mother’s care for the baby. It is the constancy of good mothering that allows a child to experience “a continuity of being” that underpins his feeling of being real and being always the same through his diverse experiences. This feeling is the basis of the “self”; it is like the center of gravity [56], [57], [58]. In this way, the importance of consistency and continuity in the antenatal care of the obese pregnant woman in relation to the healthcare team can be understood, by analogy, in the function of the baby’s experience in relation to his mother, as described by Winnicott, so that these women become self-reliant and weave their lives based on the locus of internal control.

Up to this point, this study shows a relative similarity with current studies about the emotional meanings present in the pregnancy of obese women, as well as the psychological aspects evaluated in these women. However, the results of the present study bring new contributions, pointing to fear/guilt as the most important factor responsible for the behavioral changes in the interviewees, that can be better understood and discussed in light of classic psychodynamic literature.

In many languages, fear finds an extensive vocabulary of meanings and expressions such as anguish, hate, anxiety, fright. For the interviewees, the emotional meanings referred to *vis-à-vis* their motivation to achieve the reported changes and to maintain an adequate weight gain within what is recommended were related to feelings of guilt and fear, derived from the idea of harming the loved object-child.

For Melanie Klein [59], [60], feelings of fear and guilt are closely connected and are part of the development of human emotional life. According to the author, depressive anxiety is what connects to the fear of the non-survival of the loved object. The fear of losing the loved object and the yearning to win it back, even in fantasy, is an important driving force for guilt and for the attempt to repair the damage [59], [60]. In this sense, it is possible to understand that the interviewees’ fear and guilt generates the possibility to repair possible damage caused by the obesity through the adoption of new health habits and care. However, it is important to point out that the experience in regard to fear and guilt may not necessarily be positive. As stated by Furber [20], when the obese pregnant woman deals with a health care team who reinforce the stigmas of obesity during the pregnancy, she ends up having feelings of aversion, shame about her body and self-blame.

Defending oneself against the idea of death is a constant in the nature of the human psyche [61]. The mobilizing energy created by the interviewees may be understood in light of life and death instincts. Melanie Klein [59] postulates that human beings already have them from the beginning of their lives. The author states that once we consider the existence of a death instinct, we must also consider that in the deepest layers of the mind there is a reaction to this instinct, a fear of annihilation of life. Moreover, this internal work is responsible for the first anxieties and the struggle between the instincts persists throughout life, being present in all anxiety situations. For the author, distress always happens in a connected situation and defensive strategies are used to protect oneself from a danger that is felt internally and has meanings. The author understands distress as a factor that drives mental development. A sufficient quantity of distress is needed to form symbols and fantasies. The capacity to tolerate distress, according to Klein, is the condition for the contact with, and expansion of, the mental world. [60], [62].

To get close to the gravid and obese body also involves the possibility of touching its anxieties and fears, thus opening a greater possibility of controlling anxieties and satisfying the body’s needs more appropriately.

### Limitations of the study

One of the limitations of this study is that different levels of obesity were not considered. Besides this, the pre-pregnancy IMC was calculated with the data declared by the interviewees. With regards the evaluation of the health care team, there is a possibility that the interviewees omitted negative criticism about the health care team, simply because the interviews were conducted within the service

It is worth noting that since this is a qualitative research, the results of this study are not universal and refer only to the population studied within a specific context. The findings from the emotional experiences of the obese pregnant women who had an adequate gestational weight variation support the current debate in the attempt to comprehend the phenomenon of obesity in pregnant women and to provide tools for health care teams for the antenatal care of a similar population. This observation does not prevent the recognition of the fact that the principles or concepts that this research supports may be useful in other circumstances

This research followed the Consolidated criteria for reporting qualitative research (COREQ checklist) [63] and also took into consideration the benchmarks proposed by Lincoln and Guba [64] to ensure the trustworthiness of our qualitative research.

## Conclusions

The connection between the emotional experiences of pregnant women with pre-gestational obesity and the success in keeping an adequate variation of weight during while receiving prenatal care reported by the interviewees show the dimension of complexity of human phenomena in field of health.

Knowledge of the emotional meanings given by obese women to their success at maintaining adequate weight registered throughout their pregnancy contributes to the development of practices that can promote maternal health and especially, the adherence to treatments recommended by the health care team. It is important to offer a perspective, to hear them out and to support them in a manner that fully understands and cares for these women, including the care of psychological aspects during the pregnancy.

In view of this, we suggest that more studies be carried out that take into account the perspective of the obese pregnant woman as well as studies that reveal the emotional experiences of these women with regards obesity in the puerperium/post-natal period. Besides this, we would like to recommend developing studies from the perspective of the health care team as to their feelings and strategies during the antenatal care of a woman with obesity.

## Supporting Information

S1 Fig Categories of the experiences of obese expectant mothers in relation to an adequate weight variation during pregnancy. (DOCX)

S2 Fig COREQ Checklist. (DOCX)
