# Supplementary material for: Emotional Experiences of Obese Women with Adequate Gestational Weight Variation: A Qualitative Study
Source: PLoS One. 2015 Nov 3;10(11):e0141879. doi: 10.1371/journal.pone.0141879 (PMC4631528; doi:10.1371/journal.pone.0141879)
Supplement: S1 Fig — (DOCX) [file pone.0141879.s001.docx]

| **Categories** | **Summary** | **Main themes** |
| --- | --- | --- |
| 1) The obese pregnant woman begins to think about her body | Aspects are considered of how pregnancy allowed these women to become aware of their obesity and of the importance of self-care through adopting new behavior. | - Association between the desire and act of eating with their emotions ; - Integration between mind and body; - Weight control and adherence to diet; |
| 2) The challenge of diet for the obese expectant mother | The challenge of the diet arises from the fact that the expectant mothers see themselves as being responsible for their own self-care while addressing subjective questions as to what leads them to eat. | - The body as a means of expressing desires, dissatisfactions and contradictions; - The possibility of assuming responsibility for events in their lives; |
| 3) The relation between the obese expectant mother and the professional of the antenatal healthcare team | The relation with the team was perceived to be one of attention and care. The team can be a facilitator in the process of change in the life of the obese expectant mother. | - a routine of consultations, exams, admittance to hospital and concerns about weight gain is perceived as a sign of attention and great care; - looking at themselves and facing their anxieties gives them support in their hesitations and difficulties. |
| 4) The potentializing factors for change: the motivations of the obese woman when pregnant. | Fear of causing harm to herself or to the baby proved to be the most important factor in motivating these women to adopt new eating habits and develop self-care. | - Fear of death - Fear, guilt and avoidance - Motivation for a new lifestyle |

Figure 1: Categories of the experiences of obese expectant mothers in relation to an adequate weight variation during pregnancy
